# Supplementary material for: Predicting visual function by interpreting a neuronal wiring diagram
Source: Nature. 2024 Oct 2;634(8032):113–23. doi: 10.1038/s41586-024-07953-5 (PMC11446822; doi:10.1038/s41586-024-07953-5)
Supplement: Supplementary file 6 — CRF and ERF predictions for individual TmY4 and TmY9 cells. Analogous to Supplementary Data 3, but for TmY target types. Shown are the top four monosynaptic pathways, the strongest pathway passing through each of the top ten intermediary types (ranking from Extended Data Fig. 7), and the trisynaptic pathway Tm1–TmY–Dm3–TmY (see the section entitled Prediction of spatial normalization). [file 41586_2024_7953_MOESM6_ESM.zip › DataS4/TmY9qΓèÑ/720575940613842659.pdf]

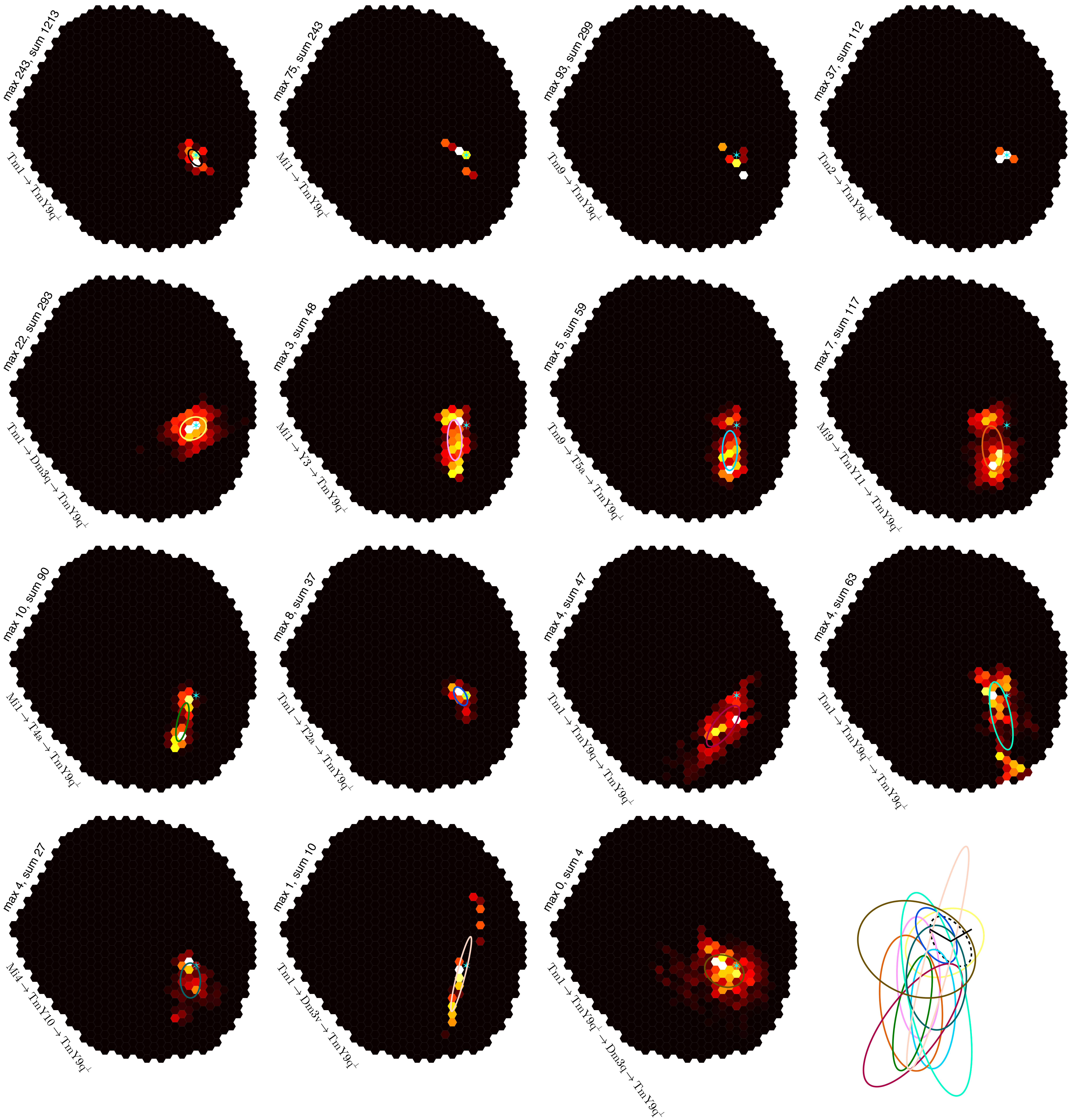

max 243, sum 1213
Tm1 → TmY9q⊥
max 75, sum 243
Mi1 → TmY9q⊥
max 93, sum 299
Tm9 → TmY9q⊥
max 37, sum 112
Tm2 → TmY9q⊥
max 22, sum 293
Tm1 → Dm3q → TmY9q⊥
max 3, sum 48
Mi1 → Y3 → TmY9q⊥
max 5, sum 59
Tm9 → T5a → TmY9q⊥
max 7, sum 117
Mi9 → TmY11 → TmY9q⊥
max 10, sum 90
Mi1 → T4a → TmY9q⊥
max 8, sum 37
Tm1 → T2a → TmY9q⊥
max 4, sum 47
Tm1 → TmY9q⊥ → TmY9q⊥
max 4, sum 63
Tm1 → TmY9q⊥ → TmY9q⊥
max 4, sum 27
Mi4 → TmY10 → TmY9q⊥
max 1, sum 10
Tm1 → Dm3v → TmY9q⊥
max 0, sum 4
Tm1 → TmY9q⊥ → Dm3q → TmY9q⊥
